# The *odd-1(tm848)* mutation has no significant effect on brood size in *Caenorhabditis elegans*

**DOI:** 10.17912/micropub.biology.000450

**Published:** 2021-09-01

**Authors:** Gabriella M. Scoca, Amy C. Groth

**Affiliations:** 1 Eastern Connecticut State University

## Abstract

The genome of *Caenorhabditis elegans *contains two genes of the odd-skipped transcription factor family, *odd-1 *and *odd-2. *In *C. elegans, odd-1 *is expressed in the intestine. A deletion mutant (*tm848) *that removes most of the *odd-1 *gene, including all three zinc fingers, has no significant effect on brood size when compared to wild-type worms.

**Figure 1. The odd-1(tm848) mutation and its effect on brood size:**
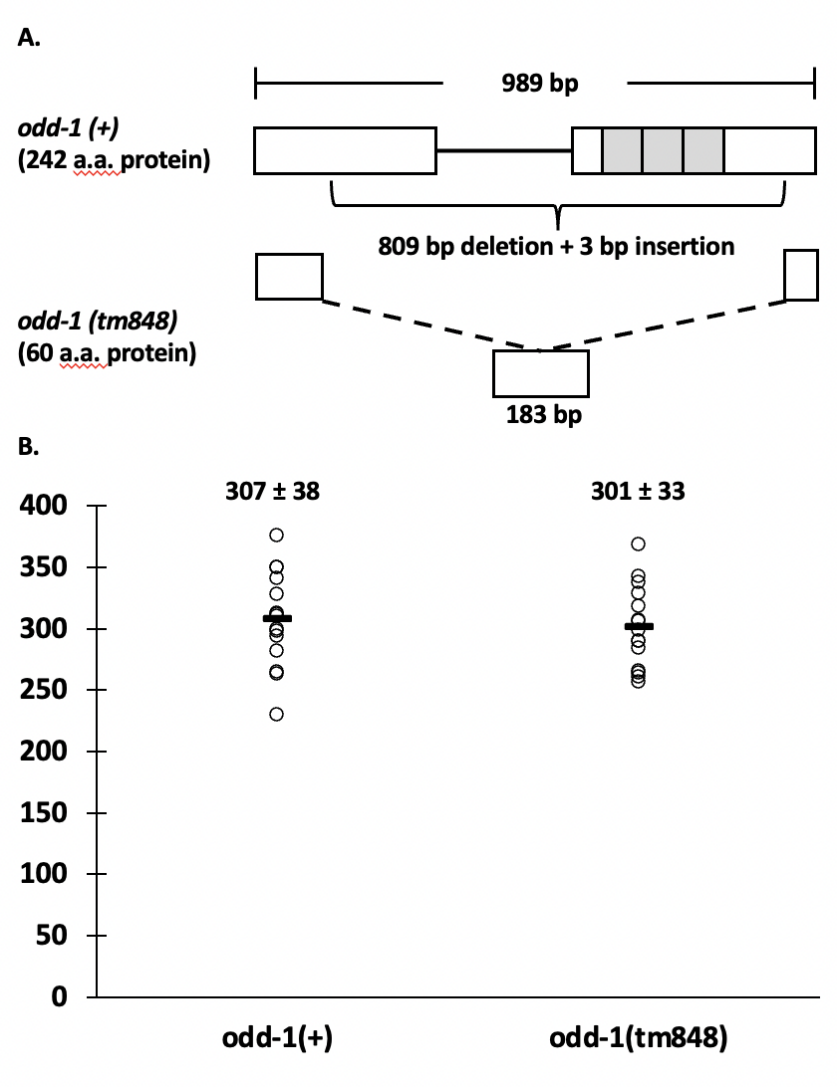
A) The 989 bp wild-type *odd-1* transcript codes for a 242 amino acid (a.a.) protein and contains two exons (white boxes), the second of which contains three zinc finger domains (gray boxes). The *odd-1(tm848)* mutation deletes most of both exons and all of the zinc fingers, and inserts one codon, resulting in a 183 bp transcript (including the stop codon) and a 60 a.a. protein. B) The brood sizes for five *odd-1(+)* and *odd-1(tm848)* worms were observed in three different experiments (a total of 15 worms were averaged for each strain). Each circle represents the brood size of an individual worm. The average is indicated on the plot by a horizontal black line. The numbers at the top of each sample group indicate the average offspring ± the standard deviation. P = 0.65 (2-tailed t-test with equal variance).

## Description

**Description**

The odd-skipped family of transcription factors are involved in a wide variety of developmental pathways across many taxa (Tena *et al.* 2007; Wang *et al.* 2005; Coulter *et al.* 1990). The genome of *C. elegans* encodes two *odd* genes, *odd-1* and *odd-2*. A deletion mutant that deletes the transcription start site of the *odd-2* gene has been classified as larval lethal by the National BioResource Project (https://nbrp.jp/en/). Early larval lethality was also seen with *odd-2* injection RNAi, due to defects of the developing gut (Buckley *et al.* 2004). Buckley *et al.* also reported larval lethality with *odd-1* injection RNAi, but only with the full-length construct that also has high homology to the zinc finger region of *odd-*2. When a smaller construct that did not contain the zinc-finger region was used, lethality was not seen (Buckley *et al.* 2004). Buckley *et al.* observed gut expression in embryos and early larvae using a 2 fragment GFP co-injection method and an integrated array. However, due to silencing of expression from multicopy arrays, it is unknown whether *odd-1* expresses in the germline. Mammals have two odd-skipped genes, odd-skipped-related 1 and 2 (OSR1 and 2). A phylogenetic tree based on the zinc finger region of the protein shows that *odd-2* is most closely related to both mammalian genes, while *odd-1* is more distantly related (Buckley *et al.* 2004). However, one of the human genes, OSR2, is most highly expressed in somatic and germline tissues related to reproduction, including uterus, vagina, cervix, Fallopian tube, ovary and testis (GTEx Consortium 2013). As a first step to clarifying the role of *odd-1* in the life cycle and development of *C. elegans*, we analyzed brood size of an *odd-1(tm848)* complex substitution (Figure 1A)*.* This substitution consists of an 809 bp deletion and a 3 bp insertion in the 989 bp *odd-1* transcript. Of the 242 amino acids in ODD-1, the N-terminal 45 amino acids are intact, as are the last 14, and one amino acid is inserted. All three predicted zinc fingers (Buckley *et al.* 2004) are deleted. This mutation is a putative null, because ODD-1 would not be able to bind DNA in a site-specific manner, although it is possible that there is a function for the remaining portion of the protein. FX848 was outcrossed five times (to create strain ACG4) and then assayed for brood size. Three brood size experiments were conducted; each recorded the brood sizes of five wild-type and five *odd-1(tm848)* worms. No significant difference was found in brood size (P=0.65), with an average wild-type brood size of 307 ±38 and an average mutant brood size of 301 ±33 (Figure 1B). The number of offspringin *odd-1* mutant worms is comparable to that of wild-type worms, indicating no obvious phenotypes affecting gamete production, egg laying or embryonic development. Additionally, no obvious early larval lethality was seen during the course of the brood size experiments, as the offspring counted on day three were generally late larvae/early adults. While low levels of lethality may be uncovered by performing a dedicated lethality assay, *odd-1(tm848)* does not appear to cause widespread early larval lethality, as was reported for injection RNAi of full-length *odd-1* (Buckley *et al.* 2004).

## Methods

**Methods**

The strain FX848 was obtained from the National BioResource Project and outcrossed five times. Primers AG382 (ATGACACTTCCATGGAATACCTTTC) and AG383 (TCAAATAGTAGTTACATCGATCAATGGC) were used for genotyping, yielding a 989 bp wild-type band and a 180 bp deletion band. Outcrossing was performed as follows. Homozygous FX848 worms were crossed to wild-type males. Individual male offspring were singled onto plates with wild-type L4 hermaphrodites. After offspring were seen, the male parents were genotyped to verify that they carried the mutation. In the first round of outcrossing, all of the male offspring were heterozygous; after the first round, only the plates with worms carrying the mutation were utilized. This process was repeated four more times, for a total of five outcrosses, at which point hermaphrodites were singled and genotyped once they had laid offspring. From hermaphrodites that were heterozygous for the mutation, offspring were singled and genotyped. One of the offspring that was identified as homozygous for the deletion was frozen as strain ACG4 (FX848 outcrossed 5x). The mutation was verified by amplification and sequencing using the primers AG356 (GTTCTTATTGGTTTTCTCCACTCTTTAAAATAGC) and AG382. For brood size assays, five wild-type and five ACG4 L4 worms were singled and moved to new plates every 24 hours for five days. Offspring on each plate were counted three days after the worms were initially placed on the plate. The experiment was repeated three times. The P-value was determined by a 2-tailed t-test with equal variance.

## Reagents

Strains

N2: wildtype

FX848: *odd-1(tm848)*

ACG4: *odd-1(tm848)* outcrossed 5X
